# Novel and Highly Efficient Carboxylative Cyclization of CO_2_ to 2-Oxazolidinones Using Nano-SiO_2_-Supported Ionic Liquid Sustainable Catalysts

**DOI:** 10.3390/molecules30030633

**Published:** 2025-01-31

**Authors:** Yulin Hu, Zongyan Tang, Xiaobing Liu

**Affiliations:** 1 College of Chemistry and Chemical Engineering, Anshun University, Anshun 561000, China; ylhanshun@126.com (Y.H.);; 2 College of Chemistry and Chemical Engineering, Jinggangshan University, Ji’an 343009, China

**Keywords:** CO_2_ utilization, supported ionic liquids, propargylic amines, 2-oxazolidinones, carboxylative cyclization reaction

## Abstract

The conversion of CO_2_ into 2-oxazolidinones through carboxylative cyclization with propargylic amines is considered an effective method for utilizing waste gas as a sustainable C1 resource and mitigating the greenhouse effect. In this context, a series of nano-SiO_2_-supported ionic liquids have been prepared and developed as multifunctional heterogeneous catalysts in the carboxylative cyclization of propargylic amines with CO_2_. The catalyst IL-SbF_6_@nano-SiO_2_ demonstrated a high compatibility with various propargylic amines, achieving excellent yields (90~98%) for the desired 2-oxazolidinones under mild conditions. Additionally, IL-SbF_6_@nano-SiO_2_ can be easily separated from the reaction mixture and reused for up to six cycles without any significant activity loss. This is important for sustainable chemistry, as it reduces waste and potentially lowers costs. This study offers novel insights into the development and design of green and efficient catalysts for the synthesis of 2-oxazolidinones from carbon dioxide.

## 1. Introduction

The use of carbon dioxide (CO_2_) as an inexpensive, non-toxic, renewable, and non-flammable C1 source for the chemical construction of valuable chemicals has excellent potential from the perspective of source employment and environmental conservancy [1,2,3,4,5,6]. Among various CO_2_ chemical fixation approaches, the carboxylative cyclization reaction between propargylic amines and CO_2_, resulting in the formation of 2-oxazolidinones, has garnered significant research interest [7]. This is primarily due to the 100% atom economy of the carboxylative cyclization reaction and the extensive applications of target products 2-oxazolidinones in chemical intermediates, pharmaceuticals, and auxiliaries [8]. In this regard, numerous catalytic systems, such as AgNO_3_/DBU [9], ZnCl_2_(TBD)_2_ [10], CuI/DBU [11], MOFs [12,13,14,15], Ag@TpPa-1/DBU [16], COFs [17,18,19], Pd(II)@bpy-CTF [20], MCM-41 [21], and dendritic NHC–gold(I) complex [22], have been explored for the carboxylative cyclization of propargylic amines and CO_2_. Nevertheless, most catalytic systems use bases and toxic solvents while requiring high gas pressure, which inevitably impedes their practical application to a certain extent. Therefore, the development of novel and efficient catalysts for the chemical fixation of CO₂ into 2-oxazolidinones under mild conditions remains a highly sought-after goal.

**Scheme 1:**
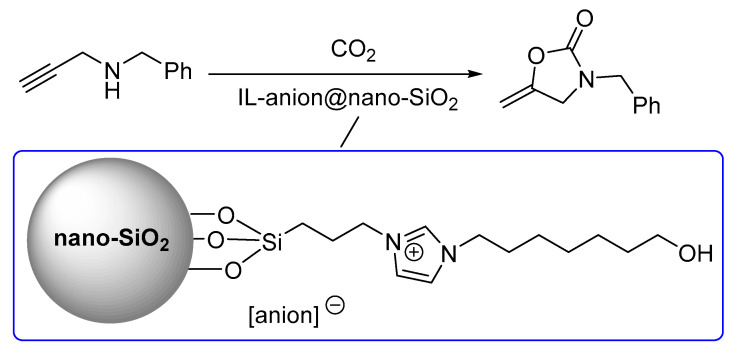
Catalytic synthesis of 2-oxazolidinones.

Ionic liquids (Ils), which are low-melting salts with customizable cations and active nucleophilic anions, have been described as green solvents with significant advantages over many other catalysts, primarily due to their extraordinary activity and selectivity, good thermostability, low vapor pressure, and non-flammability [23,24,25,26,27]. By modifying their cations and/or anions, Ils can serve as efficient catalysts for the capture and conversion of CO_2_ into 2-oxazolidinones [28,29,30]. However, Ils encounter challenges such as catalyst separation and high viscosity, which inevitably impede their practical application to a certain extent. In this regard, there is increasing interest in the heterogenization of various Ils. Ils can be immobilized onto various carriers such as zeolite, mesoporous silica, and porous carbon material, etc., which possess the benefits of sufficient contact and easy separation of heterogeneous catalysis for CO_2_ transformation [31,32,33,34]. Compared with inorganic and organic carriers nano-SiO_2_ nanoparticles possess unique structural features such as their large surface area, well-defined surface, low price, and appropriate thermal and mechanical properties, and are an ideal type of solid carrier for the immobilization of Ils [35,36,37,38]. The literature clearly indicates that silica-supported, imidazolium-based Ils could serve as effective heterogeneous catalysts in CO_2_ conversions. However, compared to homogeneous Ils, most heterogeneous counterparts generally show inferior reaction conditions and conversions. With this in mind, there remains a significant demand for developing highly efficient heterogeneous catalysts that can match the catalytic performance of homogeneous imidazolium-based Ils. Herein, a series of nano-SiO_2_-supported ionic liquids were synthesized via a covalent bond (https://www.sciencedirect.com/topics/pharmacology-toxicology-and-pharmaceutical-science/covalent-bond, URL (accessed on 29 January 2025) linkage method. The as-developed nanocomposite catalysts were explored as the heterogeneous catalysts for the carboxylative cyclization reaction of propargylic amines and CO_2_, providing excellent activity to produce 2-oxazolidinones under mild conditions (Scheme 1).

## 2. Results and Discussion

**Figure 1:**
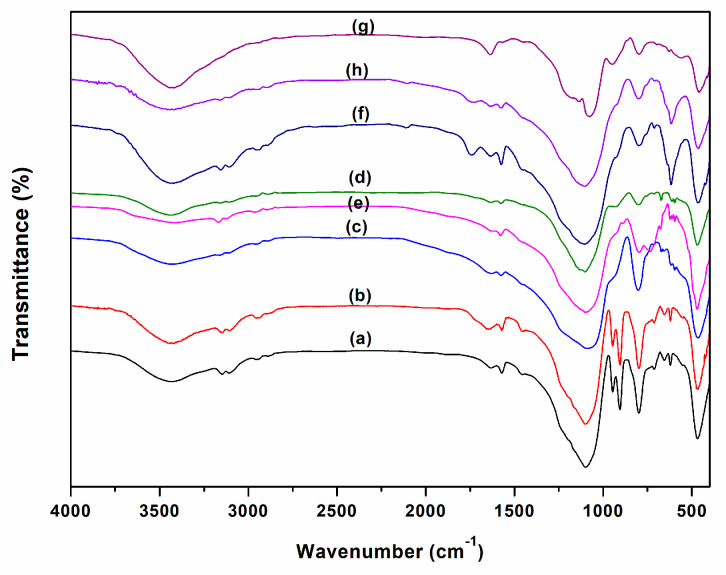
FT-IR spectra of (**a**) IL−MoO_4_@nano−SiO_2_, (**b**) IL−OH@nano−SiO_2_, (**c**) IL−HOCH_2_COO@nano−SiO_2_, (**d**) IL−BF_4_@nano−SiO_2_, (**e**) IL−SbF_6_@nano−SiO_2_, (**f**) IL−CH_3_COO@nano−SiO_2_, (**g**) IL−AlO_2_@nano−SiO_2_, and (**h**) IL−HCO_3_@nano−SiO_2_.

The supported ionic liquid nanocomposites were prepared via the reaction of 7-(1H-imidazol-1-yl)heptan-1-ol and (3-chloropropyl) triethoxysilane, followed by the anion functionalization of ionic liquid with sodium salts, and the further immobilization of functionalized ionic liquid on nano-SiO_2_ via a grafting method, and the procedure for the preparation of supported ionic liquids was shown in Scheme S1. The synthesized supported ionic liquid nanocomposites were characterized using FT-IR spectra (Figure 1). The samples exhibited absorption peaks at about 3540–3280 cm^−1^ and 972–967 cm^−1^, attributed to the stretching vibration of the –OH. The absorption peaks at about 1062–1045 cm^−1^ indicated the presence of a Si–O–Si bond. The stretching vibration peaks corresponding to the CH_3_ and CH_2_ groups were observed at around 2962–2847 cm^−1^. The absorption peaks at about 1637–1633 cm^−1^ and 1257–1251 cm^−1^ correspond to the C=C and C–N stretching vibrations of the imidazole ring, respectively [23,26,27]. The peaks at about 726–724 cm^−1^ were attributed to the bending vibrations of CH_2_ units. Additionally, the absorption peaks at around 841 cm^−1^, 678 cm^−1^, 692 cm^−1^, and 746 cm^−1^ were attributed to the Mo-O, B-F, Sb-F, and Al-O stretching vibrations of MoO_4_, BF_4_, SbF_6_, and AlO_2_ anions, respectively [39,40,41,42]. The above results confirmed the successful immobilization modification of ionic liquid on the silica support.

**Figure 2:**
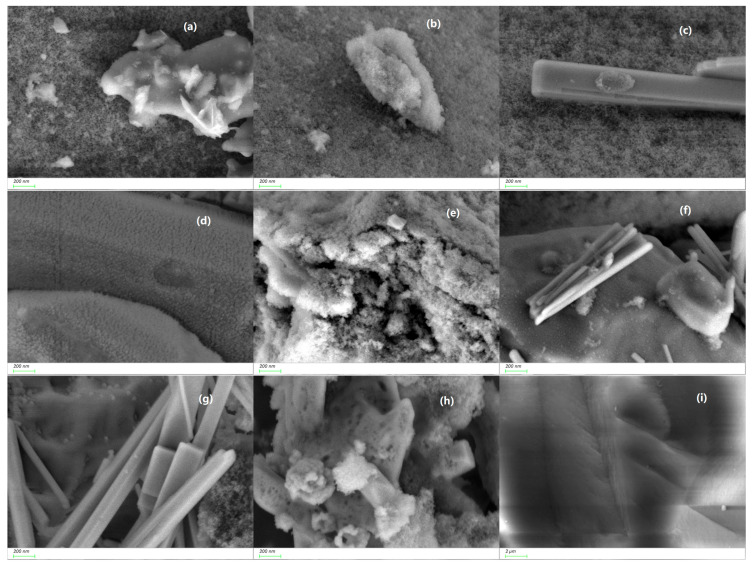
SEM images of (**a**) IL−MoO_4_@nano−SiO_2_, (**b**) IL−OH@nano−SiO_2_, (**c**) IL−HOCH_2_COO@nano−SiO_2_, (**d**) IL−BF_4_@nano−SiO_2_, (**e**) IL−SbF_6_@nano−SiO_2_, (**f**) IL−CH_3_COO@nano−SiO_2_, (**g**) IL−AlO_2_@nano−SiO_2_, (**h**) IL−HCO_3_@nano−SiO_2_, and (**i**) nano−SiO_2_.

The morphologies of the supported nanocomposites were characterized by scanning electron microscopy (SEM) (Figure 2). Compared with the surface of the original nano-SiO_2_ (Figure 2i), that of all the functionalized supported ionic liquid nanocomposites (Figure 2a–h) appeared rougher because of surface swelling during the modification. These structures resulted from the tight aggregation of irregular particles, with sizes ranging from several hundred nanometers to a few micrometers. In addition, the incorporation of functional ionic liquid groups has not caused significant structural damage to the silica support, indicating their remarkable mechanical stability. Additionally, the element mapping image (EDS) (Figure S1) demonstrated the uniform dispersion of C, N, O, Si, Mo, B, Sb, Al, and F throughout the structure of these supported ionic liquid nanocomposites, indicating the even distribution of ionic liquid units within the framework. Furthermore, the XRD spectra of the supported ionic liquid nanocomposites are presented in Figure S2. Diffraction peaks corresponding to the (02-2) plane of amorphous silica were observed at 22–25° for all samples (JCPDS Card No. 39-1425). The distinct diffraction peaks emerged at 2*θ* = 9.2° and 28.5°, corresponding to the (100) and (210) crystal planes of the Mo-O, respectively, (JCPDS Card No. 21-0569). Additionally, the characteristic diffraction peaks emerged at 2*θ* = 20.3°, 28.2°, 32.3°, 37.8°, and 47.1°, corresponding to the crystal planes of Al-O (JCPDS Card No. 10-0425). Conversely, for all the supported organic ionic liquid nanocomposites, we did not observe signals of organic ionic liquids, suggesting the uniform and low-amount ratio dispersion of these functionalized ionic liquids in silica frameworks.

The as-prepared supported ionic liquid catalysts were applied in the carboxylative cyclization of *N*-benzylprop-2-yn-1-amine with CO_2_ for the production of 3-benzyl-5-methyleneoxazolidin-2-one (Table 1). Different catalysts were tested to demonstrate the effectiveness of supported ionic liquids in this carboxylative cyclization (Table 1, entries 1–8), and the results indicated that the catalyst IL-SbF_6_@nano-SiO_2_ has superior catalytic activity, with a 97% product yield (Table 1, entry 5) compared to other supported catalysts. Additionally, the catalytic performance of bulk ionic liquids or nano-SiO_2_ support and the catalysis efficiency of the carboxylative cyclization were also evaluated. Under the same catalytic conditions, the bulk ionic liquids (IL-MoO_4_, IL-SbF_6_, IL-AlO_2_) exhibited significantly lower activity (Table 1, entries 9–11), and the reaction did not proceed in the presence of nano-SiO_2_ support (Table 1, entry 12). The results also demonstrated that there were no 2-oxazolidinone products in the absence of any catalysts (Table 1, entry 13). Notably, IL-SbF_6_@nano-SiO_2_ was the best catalyst for the carboxylative cyclization reaction. In addition, the amount of IL-SbF_6_@nano-SiO_2_ catalyst was optimized for the reactivity. As the catalyst amount decreased from 0.4 g to 0.1 g, 0.2 g, and 0.3 g, a notable reduction in product yield was observed (Table 1, entries 14–16). The yield reached 97% with 0.4 g of catalyst, and no significant improvement was observed with further increases in the catalyst amount (Table 1, entry 17).

**Figure 3:**
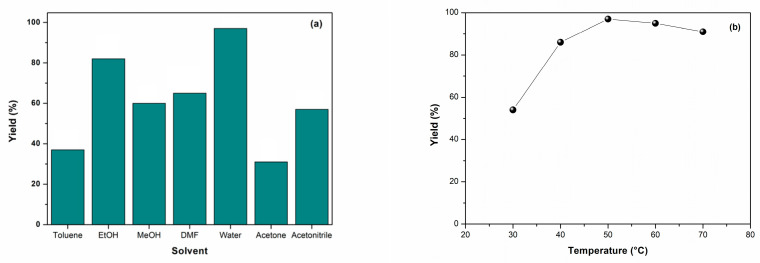

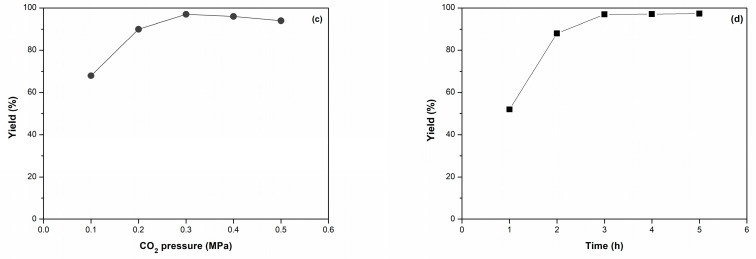
(**a**) Effect of solvent (10 mmol *N*-benzylprop-2-yn-1-amine, 0.4 g IL-SbF_6_@nano-SiO_2_, 10 mL solvent, 0.3 MPa CO_2_, 50 °C, and 3 h), (**b**) effect of temperature (10 mmol *N*-benzylprop-2-yn-1-amine, 0.4 g IL-SbF_6_@nano-SiO_2_, 10 mL H_2_O, 0.3 MPa CO_2_, and 3 h), (**c**) effect of pressure (10 mmol *N*-benzylprop-2-yn-1-amine, 0.4 g IL-SbF_6_@nano-SiO_2_, 10 mL H_2_O, CO_2_, 50 °C, and 3 h), and (**d**) effect of reaction time on the reaction (10 mmol *N*-benzylprop-2-yn-1-amine, 0.4 g IL-SbF_6_@nano-SiO_2_, 10 mL H_2_O, 0.3 MPa CO_2_, and 50 °C).

Due to the exceptional activity of IL-SbF_6_@nano-SiO_2_, it was selected as the model catalyst to optimize the reaction parameters. Figure 3a illustrates the effect of different solvents on the reaction. The influence of seven solvents, namely toluene, EtOH, MeOH, DMF, H_2_O, acetone, and acetonitrile, on the reaction, was assessed. It was revealed that the highest product yield was achieved when H_2_O was employed as the solvent. Subsequently, the influence of reaction temperature was assessed. The results, presented in Figure 3b, demonstrated significant variations in yield as the reaction temperature increased to 50 °C. According to the figure, employing 50 °C is enough to complete the reaction, and a high product yield of 97% was achieved, while lowering the temperature to 30 °C resulted in a decrease in the reaction yield. After that, with a further increase in the temperature to 70 °C, the product yield was slightly decreased due to the formation of side products. The influence of CO₂ pressure on catalytic activity was investigated. As shown in Figure 3c, increasing the reaction pressure from 0.1 to 0.3 MPa significantly enhanced the catalytic activity. Further increasing the pressure to 0.5 MPa led to a slower decrease in activity. Consequently, 0.3 MPa was identified as the optimal reaction pressure. The effect of reaction time was also examined (Figure 3d). As shown, by increasing the reaction time from 1 h to 3 h, the catalytic activity increased and reached the maximum value at 3 h, and no significant improvement was observed with further increases in reaction time.

**Figure 4:**
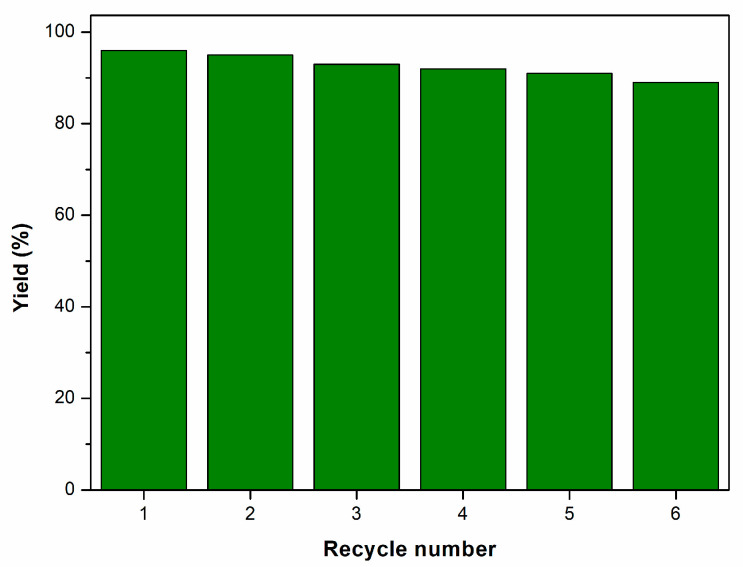
Catalytic activity of the recycled IL-SbF_6_@nano-SiO_2_ catalyst.

The thermal stability of the nano-SiO_2_@IL-SbF_6_ catalyst was determined using thermogravimetric analysis (TGA) (Figure S3). The initial weight loss of 2.77% observed below 100 °C was ascribed to the desorption of adsorbed water and solvent molecules. The major weight loss of 27.46% occurring above 150 °C was due to the thermal decomposition of the chemically grafted ionic liquid segment. TGA confirmed that the superior IL-SbF_6_@nano-SiO_2_ catalyst exhibit sufficient thermal stability for subsequent application in CO_2_ carboxylative cyclization reactions. Reusability is an important factor in the evaluation of heterogeneous catalysts. The recycling performance of IL-SbF_6_@nano-SiO_2_ was investigated using *N*-benzylprop-2-yn-1-amine as the model substrate under optimized conditions. In each cycle, the catalyst was recovered through filtration. After rinsing with ethanol and vacuum drying, it was reused in the next cycle. The results depicted in Figure 4 show that the yield was still as high as 90% even after six runs, indicating its commendable reusability. In addition, a hot filtration experiment was performed (Figure S4). The reaction was halted after 1.5 h, and the catalyst was promptly removed via hot filtration. The reaction mixture was then allowed to proceed under the same conditions for an additional five hours. It can be seen that there are no obvious conversions in the reaction, suggesting the heterogeneous features and good stability of the IL-SbF_6_@nano-SiO_2_ catalyst. To perform a thorough evaluation of the reusability and stability of the catalyst, the reused catalyst nano-SiO_2_@IL-SbF_6_ was subjected to characterization using FT-IR (Figure S5) and XPS spectroscopy (Figure S6) after six runs, compared with those of the fresh IL-SbF_6_@nano-SiO_2_. The recovered sample exhibited all the original characteristic peaks, which indicated that the chemical structure and composition of the reused IL-SbF_6_@nano-SiO_2_ did not change. SEM images (Figure S7) also revealed no significant changes in morphology. Therefore, the novel supported catalyst has excellent stability and recyclability for CO_2_ carboxylative cyclization.

To explore the applicability scope of IL-SbF_6_@nano-SiO_2_, various propargylic amines were employed under optimized conditions (Table 2). The results showcase that a series of propargylic amines with electron-donating and electron-withdrawing groups could be converted into the respective products in excellent yields (94–98%) (Table 2, entries 2–7). Interestingly, other propargylic amines of *N*-(thiophen-2-ylmethyl)prop-2- yn-1-amine, *N*-(prop-2-yn-1-yl)butan-1-amine, and *N*-(prop-2-yn-1-yl)cyclohexanamine were also efficiently converted to their corresponding products in excellent yields (90–97%) (Table 2, entries 8–10). These findings provide additional evidence of the effective catalytic performance of the heterogeneous supported catalyst.

**Scheme 2:**
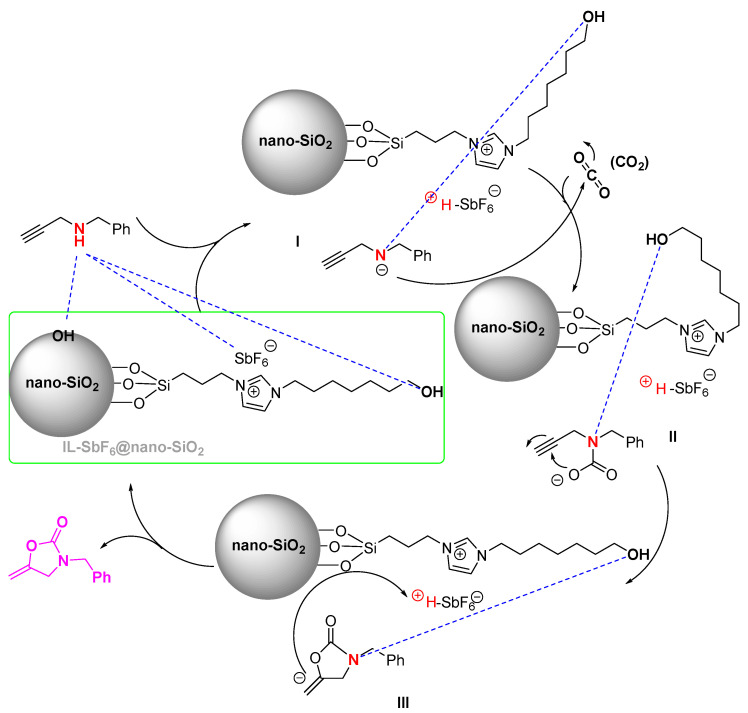
Possible mechanism of carboxylative cyclization.

In light of the experimental data and literature references [11,12,13,14,15,16], a possible reaction mechanism for the carboxylative cyclization of CO_2_ with propargylic amines, catalyzed by IL-SbF_6_@nano-SiO_2_, was proposed and depicted in Scheme 2. Initially, the OH groups and SbF_6_ of the nanocatalyst work together to activate the propargylic amines through hydrogen bonding, followed by the deprotonation of the N-H bond to form the intermediate **I**. The outstanding catalytic performance of IL-SbF_6_@nano-SiO_2_ may be attributed to the synergistic effect of the two active sites. Next, the intermediate **I** carries out an electrophilic attack on propargylic amines by CO_2_, forming a carbamate intermediate **II**. Subsequently, intramolecular cyclization ensues, wherein the negatively charged oxygen in the carbamate triggers an attack on the triple bond to form the intermediate **III**. This sequence culminates in the generation of the final product via the protonation process, thereby regenerating the catalyst and completing the catalytic cycle.

## 3. Materials and Methods

The materials and methods section of materials, synthesis of catalysts, and catalytic carboxylative cyclization is shown in the Supplementary Materials.

## 4. Conclusions

In summary, a type of nano-SiO_2_-supported ionic liquid heterogeneous catalyst was developed to catalyze the carboxylative cyclization of propargylic amines with CO_2_ to produce 2-oxazolidinones. Among the synthesized supported ionic liquids, IL-SbF_6_@nano-SiO_2_ showed the better catalytic property, and the reactions could proceed smoothly under mild conditions to obtain 2-oxazolidinones products in excellent yields. As a highly active and recyclable catalyst, IL-SbF_6_@nano-SiO_2_ effectively facilitated the CO_2_ carboxylative cyclization reaction under mild conditions and demonstrated excellent cycling performance, maintaining its catalytic activity over multiple cycles, with a negligible loss of activity. The green protocol is attractive in terms of the simplicity of the procedure, easy separation of the catalyst, environmental compatibility, high yields, recycle exploitation, and excellent isolated yields. This study offers a promising avenue for converting CO_2_ into high-value-added chemicals, representing a sustainable alternative to conventional methods. 

## Figures and Tables

**Table 1:** Catalyst screening for carboxylative cyclization ^a^.

Entry	Catalyst	Catalyst (g)	Time (h)	Yield (%) ^b^
1	IL−MoO_4_@nano−SiO_2_	0.4	3	84
2	IL−OH@nano−SiO_2_	0.4	5	65
3	IL−HOCH_2_COO@nano−SiO_2_	0.4	5	60
4	IL−BF_4_@nano−SiO_2_	0.4	5	70
5	IL−SbF_6_@nano−SiO_2_	0.4	3	97
6	IL−CH_3_COO@nano−SiO_2_	0.4	6	56
7	IL−AlO_2_@nano−SiO_2_	0.4	3	80
8	IL−HCO_3_@nano−SiO_2_	0.4	3	51
9	IL−MoO_4_	0.4	3	82
10	IL−SbF_6_	0.4	3	87
11	IL−AlO_2_	0.4	3	79
12	nano−SiO_2_	0.5	24	trace
13	none	0	24	0
14	IL−SbF_6_@nano−SiO_2_	0.1	5	49
15	IL−SbF_6_@nano−SiO_2_	0.2	5	67
16	IL−SbF_6_@nano−SiO_2_	0.3	3	88
17	IL−SbF_6_@nano−SiO_2_	0.5	3	97

^a^ Reaction conditions: *N*-benzylprop-2-yn-1-amine (10 mmol), H_2_O (10 mL), CO_2_ (0.3 MPa), and 50 °C. ^b^ Isolated yield.

**Table 2:** Catalytic synthesis of various 2-oxazolidinones with IL-SbF_6_@nano-SiO_2_ ^a^.

Entry	Substrate	Product	Time (h)	Yield (%) ^b^
1	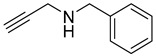	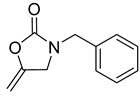	3	97
2	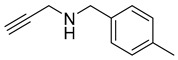	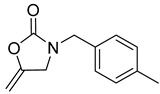	3	96
3	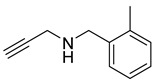	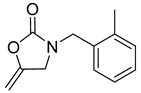	3	96
4	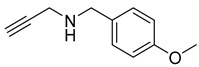	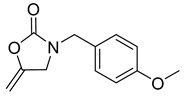	3	98
5	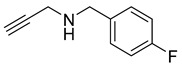	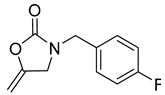	3	98
6	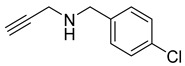	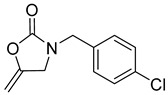	3	96
7	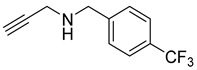	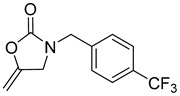	3	94
8	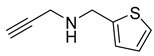	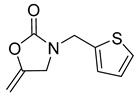	3	97
9	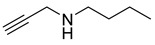	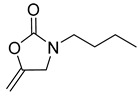	4	92
10	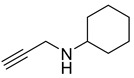	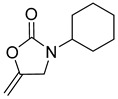	4	90

^a^ Reaction conditions: propargylic amine (10 mmol), IL-SbF_6_@nano-SiO_2_ (0.4 g), H_2_O (10 mL), CO_2_ (0.3 MPa), and 50 °C. ^b^ Isolated yield.

## Data Availability

The authors confirm that all the data needed to support this study are presented within the article.

## Supplementary Materials

The following supporting information can be downloaded at: https://www.mdpi.com/article/10.3390/molecules30030633/s1. Scheme S1: Preparation of supported ionic liquids; Figures S1 and S2: EDS images, XRD diffractograms of supported ionic liquids, respectively; Figure S3: TGA curve of IL-SbF_6_@nano-SiO_2_ catalyst; Figure S4: Hot filtration test during the catalytic process; Figure S5: FT-IR spectra of IL-SbF_6_@nano-SiO_2_ catalyst before and after reaction; Figure S6: XPS spectra of fresh and reused IL-SbF_6_@nano-SiO_2_ catalyst; and Figure S7: SEM images of fresh and six times recycled IL-SbF_6_@nano-SiO_2_ catalyst.

## References

[1] Sun Z., Liao Y., Zhao S., Zhang X., Liu Q., Shi X. (2022). Research progress in metal–organic frameworks (MOFs) in CO_2_ capture from post-combustion coal-fired flue gas: Characteristics, preparation, modification and applications. J. Mater. Chem. A.

[2] Chen T.W., Chen S.M., Anushya G., Kannan R., Al-Sehemi A.G., Alargarsamy S., Gajendran P., Ramachandran R. (2023). Development of different kinds of electrocatalyst for the electrochemical reduction of carbon dioxide reactions: An overview. Molecules.

[3] Jelmy E.J., Thomas N., Mathew D.T., Louis J., Padmanabhan N.T., Kumaravel V., John H., Pillai S.C. (2021). Impact of structure, doping and defect-engineering in 2D materials on CO_2_ capture and conversion. React. Chem. Eng..

[4] Velty A., Corma A. (2023). Advanced zeolite and ordered mesoporous silica-based catalysts for the conversion of CO_2_ to chemicals and fuels. Chem. Soc. Rev..

[5] Khan M.N., Ingen Y., Boruah T., McLauchlan A., Wirth T., Melen R.L. (2023). Advances in CO_2_ activation by frustrated Lewis pairs: From stoichiometric to catalytic reactions. Chem. Sci..

[6] Baran T., Caringella D., Dibenedetto A., Aresta M. (2024). Pitfalls in photochemical and photoelectrochemical reduction of CO_2_ to energy products. Molecules.

[7] Arshadi S., Vessally E., Sobati M., Hosseinian A., Bekhradnia A. (2017). Chemical fixation of CO_2_ to N-propargylamines: A straightforward route to 2-oxazolidinones. J. CO_2_ Util..

[8] Wang B., Guo Z., Wei X. (2023). Recent advances on oxazolidinones synthesize from carbon dioxide. J. Fuel Chem. Technol..

[9] Yoshida M., Mizuguchi T., Shishido K. (2012). Synthesis of oxazolidinones by efficient fixation of atmospheric CO_2_ with propargylic amines by using a silver/1,8-diazabicyclo [5.4.0]undec-7-ene (DBU) dual-catalyst system. Chem. Eur. J..

[10] Liu X., Wang M.Y., Wang S.Y., Wang Q., He L.N. (2017). In situ generated zinc(II) catalyst for incorporation of CO_2_ into 2-oxazolidinones with propargylic amines at atmospheric pressure. ChemSusChem.

[11] Zhao Y., Qiu J., Tian L., Li Z., Fan M., Wang J. (2016). New copper(I)/DBU catalyst system for the carboxylative cyclization of propargylic amines with atmospheric CO_2_: An experimental and theoretical study. ACS Sustain. Chem. Eng..

[12] Wu Z.L., Zhai Y.T., Bian G.G., Guo L.J., Zhang Y.X., Wei H.Y. (2024). Conversion of propargylic amines with CO_2_ catalyzed by a highly stable copper(I) iodide thorium-based heterometal–organic framework. Inorg. Chem..

[13] Zhang C.H., Hu T.D., Zhai Y.T., Zhang Y.X., Wu Z.L. (2023). Stepwise engineering of the pore environment within metal–organic frameworks for green conversion of CO_2_ and propargylic amines. Green Chem..

[14] Ding M., Flaig R.W., Jiang H.L., Yaghi O.M. (2019). Carbon capture and conversion using metal–organic frameworks and MOF-based materials. Chem. Soc. Rev..

[15] Giri P.K., Parihar V., Kumar S., Nagaraja C.M. (2024). Copper nanoparticles anchored on the metal–organic framework as recyclable catalyst for CO_2_ fixation to high-value compounds. ACS Appl. Nano Mater..

[16] Ghosh S., Molla R.A., Kayal U., Bhaumik A., Islam S.M. (2019). Ag NPs decorated on a COF in the presence of DBU as an efficient catalytic system for the synthesis of tetramic acids via CO_2_ fixation into propargylic amines at atmospheric pressure. Dalton Trans..

[17] Zhang Y., Li H., He X., Wang A., Bai G., Lan X. (2023). Covalent organic frameworks embedding single cadmium sites for efficient carboxylative cyclization of CO_2_ with propargylic amines. Green Chem..

[18] Ghosh S., Khan T.S., Ghosh A., Chowdhury A.H., Haider M.A., Khan A., Islam S.M. (2020). Utility of silver nanoparticles embedded covalent organic frameworks as recyclable catalysts for the sustainable synthesis of cyclic carbamates and 2-oxazolidinones via atmospheric cyclizative CO_2_ capture. ACS Sustain. Chem. Eng..

[19] Qiu J., Qi X., Zhu K., Zhao Y., Wang H., Li Z., Wang H., Zhao Y., Wang J. (2024). Cu^I^-anchored porous covalent organic frameworks for highly efficient conversion of propargylic amines with CO_2_ from flue gas. Green Chem..

[20] Kishan R., Rani P., Singh G., Nagaraja C.M. (2024). Functionalized Covalent Triazine Framework (CTF) for catalytic CO_2_ fixation and synthesis of value-added chemicals. Cryst. Growth Des..

[21] Matsuo H., Choi J.C., Fujitani T., Fujita K. (2020). Silica-catalyzed carboxylative cyclization of propargylic amines with CO_2_. Tetrahedron Lett..

[22] Fujita K., Inoue K., Sato J., Tsuchimoto T., Yasuda H. (2016). Carboxylative cyclization of propargylic amines with CO2 catalyzed by dendritic N-heterocyclic carbene–gold(I) complexes. Tetrahedron.

[23] Tariq M., Freire M.G., Saramago B., Coutinho J.A.P., Lopes J.N.C., Rebelo L.P.N. (2012). Surface tension of ionic liquids and ionic liquid solutions. Chem. Soc. Rev..

[24] Ab Rahim A.H., Yunus N.M., Bustam M.A. (2023). Ionic liquids hybridization for carbon dioxide capture: A review. Molecules.

[25] Więcławik J., Chrobok A. (2023). Gallium(III)- and indium(III)-containing ionic liquids as highly active catalysts in organic synthesis. Molecules.

[26] McNeice P., Marr P.C., Marr A.C. (2021). Basic ionic liquids for catalysis: The road to greater stability. Catal. Sci. Technol..

[27] Zhang S.J., Lu X.M. (2006). Ionic Liquids: From Fundamental Research to Industrial Applications.

[28] Wang M.Y., Song Q.W., Ma R., Xie J.N., He L.N. (2016). Efficient conversion of carbon dioxide at atmospheric pressure to 2-oxazolidinones promoted by bifunctional Cu(II)-substituted polyoxometalate-based ionic liquids. Green Chem..

[29] Wu J., Niu J., Liu H., Xie R., Zhu N. (2024). Conversion of atmospheric CO_2_ catalyzed by thiolate-based ionic liquids under mild conditions: Efficient synthesis of 2-oxazolidinones. Org. Biomol. Chem..

[30] Vishwakarma N.K., Singh A.K., Hwang Y.H., Ko D.H., Kim J.O., Babu A.G., Kim D.P. (2017). Integrated CO_2_ capture-fixation chemistry via interfacial ionic liquid catalyst in laminar gas/liquid flow. Nat. Commun..

[31] Pappuru S., Shpasser D., Gazit O. (2023). Synthesis of polycarbonates from CO_2_ promoted by immobilized ionic liquid functionalized di-Mg complex catalyst. ChemCatChem.

[32] Nale D.B., Saigaonkar S.D., Bhanage B.M. (2014). An efficient synthesis of quinazoline-2,4(1H,3H)-dione from CO_2_ and 2-aminobenzonitrile using [Hmim]OH/SiO_2_ as a base functionalized supported ionic liquid phase catalyst. J. CO2 Util..

[33] Akbari Z., Ghiaci M. (2017). Heterogenization of a green homogeneous catalyst: Synthesis and characterization of imidazolium ionene/Br–Cl–@SiO_2_ as an efficient catalyst for the cycloaddition of CO_2_ with epoxides. Ind. Eng. Chem. Res..

[34] Martinez A.S., Hauzenberger C., Sahoo A.R., Csendes Z., Hoffmann H., Bica K. (2018). Continuous conversion of carbon dioxide to propylene carbonate with supported ionic liquids. ACS Sustain. Chem. Eng..

[35] Weiss E., Dutta B., Kirschning A., Abu-Reziq R. (2014). BMIm-PF_6_@SiO_2_ microcapsules: Particulated ionic liquid as a new material for the heterogenization of catalysts. Chem. Mater..

[36] Garcia I.M., Souza V.S., Balhaddad A.A., Mokeem L., Melo M.A.S., Scholten J.D., Collares F.M. (2024). Ionic liquid-based silane for SiO_2_ nanoparticles: A versatile coupling agent for dental resins. ACS Appl. Mater. Interfaces.

[37] Li Z., Luo A., Zhou R., Li X., Li H. (2024). Preparation of ionic liquid@SiO_2_ nanocapsules for improving self-lubricating performance of PA6 composites. Polymer.

[38] Hu F., Qi F., Xiang Z., Zhang B., Qi F., Zhao N., Ouyang X. (2021). Synergistic enhancement effect of nano-SiO_2_ and ionic liquids on mechanical properties and impact resistance of polyurethane elastomer. Compos. Commun..

[39] Kumar H., Katal A. (2018). Temperature dependent physicochemical and spectroscopic (FT-IR) studies of citrate salts (trilithium citrate and triammonium citrate) in aqueous ionic liquid [C_4_mim][BF_4_] (1-butyl-3-methyl imidazolium tetrafluroborate) solutions. J. Mol. Liq..

[40] Cascales C., Blas A.M., Rico M., Volkov V., Zaldo C. (2005). The optical spectroscopy of lanthanides R^3+^ in ABi(XO_4_)_2_ (A = Li, Na; X = Mo, W) and LiYb(MoO_4_)_2_ multifunctional single crystals: Relationship with the structural local disorder. Opt. Mater..

[41] Świetlik R., Jankowski D., Fourmigué M., Yakushi K. (2011). Infrared and Raman studies of the anion ordering transitions in paramagnetic organometallic radical cation salts [Cp_2_Mo(dmit)]X (X = PF_6_, SbF_6_). Vib. Spectrosc..

[42] Lutz W., Rüscher C.H., Heidemann D. (2002). Determination of the framework and non-framework [SiO_2_] and [AlO_2_] species of steamed and leached faujasite type zeolites: Calibration of IR, NMR, and XRD data by chemical methods. Micropor. Mesopor. Mater..

