# Supplementary material for: Novel and Highly Efficient Carboxylative Cyclization of CO2 to 2-Oxazolidinones Using Nano-SiO2-Supported Ionic Liquid Sustainable Catalysts
Source: Molecules. 2025 Jan 31;30(3):633. doi: 10.3390/molecules30030633 (PMC11820950; doi:10.3390/molecules30030633)
Supplement: Supplementary file 1 [file molecules-30-00633-s001.zip › molecules-3422245-supplementary.pdf]

Supporting Information

Novel and Highly Efficient Carboxylative Cyclization of CO_2 to 2-Oxazolidinones over nano-SiO_2 Supported Ionic Liquids Sustainable Catalysts

Yu Lin Hu [1], Zong Yan Tang [1] and Xiao Bing Liu [2,*]

[1] College of Chemistry and Chemical Engineering, Anshun University, Anshun 561000, China
[2] College of Chemistry and Chemical Engineering, Jinggangshan University, Ji'an 343009, China
* Correspondence: liuxiaobing805@163.com; ylhanshun@126.com

Materials and Methods

Materials and apparatus

All reagents and chemicals were purchased from Sigma-Aldrich. All the above materials were of analytical grade and not further purified. Fourier transform infrared spectroscopy (FT-IR) analysis were conducted using a Nicolet Nexus 470 (KBr disks). The progress of reactions was monitored by the gas chromatograph (GC, Agilent 7890A) equipped with an HPINNO Wax capillary column. X-ray diffraction (XRD) was carried out to determine crystal phases of the samples using a Rigaku Ultima IV X-ray diffractometer by employing Cu-Kα radiation. Scanning electron microscopy (SEM) characterization and energy dispersive X-ray (EDX) mapping were analyzed using a GeminiSEM 360 apparatus. The thermal stability of the synthesized materials were analyzed on a METTLER TOLEDO-TGA/DSC-1 thermogravimetric analyzer (TGA) under nitrogen atmosphere. The temperature range is 25~600 °C with a rate of 10 °C/min. X-ray photoelectron spectra (XPS) were obtained using a Thermo Scientific K-Alpha Nexsa instrument with Al Kα radiation as the X-ray source. The C 1s peak at 284.8 eV was used as the reference for the binding energies. Melting points were measured by an electro-thermal IA 9100 apparatus. ^{1}H NMR spectra were recorded on a Bruker Avance 400 MHz spectrometer, ^{13}C NMR spectra were recorded on a Bruker Avance 125 MHz spectrometer. Elemental analysis was performed on a Vario Micro cube Elemental Analyzer.

Preparation of supported ionic liquids catalysts (Scheme S1)

7-(1H-imidazol-1-yl)heptan-1-ol (0.1 mol, 18.2 g), (3-chloropropyl) triethoxysilane (0.1 mol, 24.1 g) and toluene (80 mL) were added to a round bottom flask with a condenser pipe. The mixture was vigorously stirred at 95 °C under nitrogen atmosphere for 24 h. After that, the solvent was isolated by liquid-liquid separation and the residue was washed with ether (20 mL × 3) followed by vacuum drying at 70 °C to give **1** (37.0 g, yield 87.4%). Next, Na_2MoO_4 (0.05 mol, 10.3 g), NaOH (0.05 mol, 2 g), $HOCH_2COONa$ (0.05 mol, 4.9 g), $NaBF_4$ (0.05 mol, 5.49 g), $NaSbF_6$ (0.05 mol, 12.9 g), CH_3COONa (0.05 mol, 4.1 g), $NaAlO_2$ (0.05 mol, 4.1 g) or $NaHCO_3$ (0.05 mol, 4.2 g), and **1** (0.05 mol, 21.2 g) were added into CH_3CN (60 mL) and the mixture was stirred vigorously at 50 °C for 24 h. After the reaction, the solution was removed and the crude ionic liquid was washed with ether (20 mL × 3) three times to remove unreacted substrates, followed by vacuum drying at 50 °C to give IL-anion **2** (IL-MoO_4 19.6 g, yield 71.8%; IL-OH 17.2 g, yield 85.0%; IL-$HOCH_2COO$ 18.5 g, yield 80.0%; IL-BF_4 19.0 g, yield 80.1%; IL-SbF_6 22.8 g, yield 73.2%; IL-CH_3COO 19.0 g, yield 85.1%; IL-AlO_2 18.5 g, yield 82.8%; IL-HCO_3 16.7 g, yield 74.5%). Finally, nano-SiO_2 (1.0 g) and IL-anion **II** (0.6 g), toluene (60 mL) were stirred at 110 °C for 24 h. The resulting precipitate was separated from the solution by filtration and dried under vacuum at 70 °C for 12 h to give the supported ionic liquids IL-anion@nano-SiO_2 (IL-MoO_4@nano-SiO_2 1.30 g, IL-OH@nano-SiO_2 1.34 g, IL-$HOCH_2COO$@nano-SiO_2 1.35 g, IL-BF_4@nano-SiO_2 1.37 g, IL-SbF_6@nano-SiO_2 1.39 g, IL-CH_3COO@nano-SiO_2 1.28 g, IL-AlO_2@nano-SiO_2 1.31 g, IL-HCO_3@nano-SiO_2 1.40 g).

Compound **1**: IR (cm^{-1}): 3407, 3023, 2974, 2931, 2890, 2851, 1512, 1462, 1392, 1378, 1089; Elemental analysis for $C_{19}H_{39}N_2O_4SiCl$: C, 56.21; Cl, 9.02; N, 6.15; O, 15.48; Si, 6.71.

Compound **2**:

IL-MoO_4: IR (cm^{-1}): 3502, 3027, 2971 2928, 2889, 2847, 1517, 1463, 1387, 1379, 1081, 912; Elemental analysis for $C_{19}H_{39}N_2O_8SiMo$: C, 52.33; Mo, 13.20; N, 6.03; O, 18.32; Si, 5.18.

IL-OH: IR (cm^{-1}): 3421, 3030, 2972, 2925, 2887, 2849, 1521, 1462, 1388, 1381, 1076; Elemental analysis for $C_{19}H_{40}N_2O_5Si$: C, 56.61; N, 7.15; O, 17.86; Si, 6.75.

IL-$HOCH_2COO$: IR (cm^{-1}): 3357, 3025, 2975, 2930, 2888, 2853, 1692, 1530, 1457, 1392, 1380, 1072; Elemental analysis for $C_{21}H_{42}N_2O_7Si$: C, 58.43; N, 5.97; O, 21.46; Si, 5.08.

IL-BF_4: IR (cm^{-1}): 3410, 3025, 2973, 2928, 2889, 2841, 1530, 1466, 1341, 1380, 1065, 845; Elemental analysis for $C_{19}H_{39}N_2O_4SiBF_4$: C, 55.24; B, 2.46; F, 3.78; N, 6.51; O, 16.12; Si, 5.31.

IL-SbF_6: IR (cm^{-1}): 3378, 3090, 2956, 2932, 2872, 2832, 1535, 1462, 1345, 1387, 1052, 736; Elemental analysis for $C_{19}H_{39}N_2O_4SiSbF_6$: C, 53.79; Sb, 5.11; F, 6.85; N, 4.79; O, 15.64; Si, 4.76.

IL-CH_3COO: IR (cm^{-1}): 3378, 3056, 2961, 2935, 2880, 2851, 1697, 1534, 1462, 1391, 1382, 1051; Elemental analysis for $C_{21}H_{42}N_2O_6Si$: C, 58.04; N, 6.05; O, 20.86; Si, 5.15.

IL-AlO2: IR (cm^{-1}): 3337, 3025, 2967, 2942, 2876, 2846, 1531, 1467, 1395, 1381, 1062, 685; Elemental analysis for $C_{19}H_{39}N_2O_6SiAl$: C, 54.51; Al, 9.35; N, 5.82; O, 17.76; Si, 5.73.
IL-HCO3: IR (cm^{-1}): 3402, 3033, 2932, 2871, 2842, 1852, 1647, 1528, 1463, 1389, 1384, 1037; Elemental analysis for $C_{20}H_{40}N_2O_7Si$: C, 56.42; N, 7.01; O, 17.85; Si, 6.07.

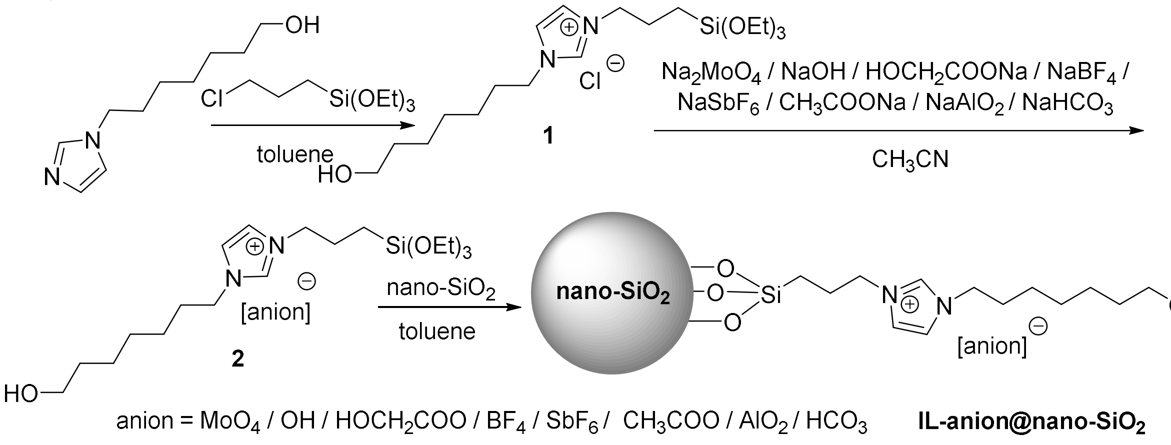

Scheme S1. Preparation of supported ionic liquids.

General procedure for the carboxylative cyclization of propargylic amines with CO_2
The carboxylative cyclization reactions were conducted in a 50 mL stainless steel autoclave equipped with a magnetic stirrer. Propargylic amine (10 mmol), IL-SbF_6@nano-SiO_2 (0.4 g), and H_2O (10 mL) were added to the autoclave. The interior air of the autoclave was replaced with CO_2 through three vacuum/CO_2 cycles. The mixture was stirred at 50 °C for the desired time and then pressurized with CO_2 of 0.3 MPa. The evaluation of the conversion and selectivity of the reaction was conducted using gas chromatograph (GC, Agilent 7890A). After completion of the reaction, the mixture was cooled to room temperature. The solid catalyst was recovered from the reaction mixture by simple filtration and washed twice with ethanol, dried under vacuum, and reused in the next cycle. The aqueous layer was then extracted using dichloromethane. The organic phase was dried and the solvent was removed. The crude product was purified by a flash column chromatography using ethyl acetate/petroleum ether (v/v = 1/8). The obtained products were known and confirmed by comparing their ^1H NMR data with the known compounds.
Characterization spectra of 2-oxazolidinones
3-benzyl-5-methyleneoxazolidin-2-one (Table 2, entry 1) [13]: White solid, 1.83 g, 97%, m.p. 52.5-54.2 °C; ^1H NMR (400 MHz, $CDCl_3$) (δ/ppm): 4.01 (m, CH_2, 2H), 4.23 (d, =CH_2, 1H), 4.46 (s, CH_2, 2H), 4.75 (d, =CH_2, 1H), 7.28-7.40 (m, Ar-H, 5H); ^{13}C NMR (125 MHz, $CDCl_3$): δ = 47.4, 48.1, 83.4, 128.2, 128.5, 129.2, 135.3, 149.4, 155.7 ppm; Elemental analysis for $C_{11}H_{11}NO_2$: C, 69.80; H, 5.83; N, 7.35; O, 16.87. Found: C, 69.83; H, 5.86; N, 7.40; O, 16.91.
3-(4-methylbenzyl)-5-methyleneoxazolidin-2-one (Table 2, entry 2) [13]: White liquid, 1.95 g, 96%; ^1H NMR (400 MHz, $CDCl_3$) (δ/ppm): 2.31 (s, CH_3, 3H), 3.98 (m, CH_2, 2H), 4.19 (d, =CH_2, 1H), 4.42 (s, CH_2, 2H), 4.72 (d, =CH_2, 1H),, 7.15-7.26 (m, Ar-H, 4H); ^{13}C NMR (125 MHz, $CDCl_3$): δ = 21.4, 47.1, 47.8, 86.8, 128.1, 129.5, 131.7, 138.3, 149.2, 155.7 ppm; Anal. Calcd. for $C_{12}H_{13}NO_2$: C, 70.86; H, 6.42; N, 6.85; O, 15.71. Found: C, 70.92; H, 6.45; N, 6.89; O, 15.74.
3-(2-methylbenzyl)-5-methyleneoxazolidin-2-one (Table 2, entry 3) [15]: White liquid, 1.95 g, 96%; ^1H NMR (400 MHz, $CDCl_3$) (δ/ppm): 3.79 (s, CH_3, 3H), 3.98 (m, CH_2, 2H), 4.20 (d, =CH_2, 1H), 4.39 (s, CH_2, 2H), 4.71 (d, =CH_2, 1H),, 6.85-6.89 (m, Ar-H, 2H), 7.16-7.24 (m, Ar-H, 2H); ^{13}C NMR (125 MHz, $CDCl_3$): δ = 19.2, 46.2, 47.5, 86.7, 126.1, 128.7, 129.4, 130.8, 132.9, 137.2, 149.1, 155.4 ppm; Elemental analysis for $C_{12}H_{13}NO_2$: C, 70.87; H, 6.42; N, 6.86; O, 15.69. Found C, 70.92; H, 6.45; N, 6.89; O, 15.74.
3-(4-methoxybenzyl)-5-methyleneoxazolidin-2-one (Table 2, entry 4) [15]: White liquid, 2.15 g, 98%; ^1H NMR (400 MHz, $CDCl_3$) (δ/ppm): 2.31 (s, CH_3, 3H), 3.98 (m, CH_2, 2H), 4.19 (d, =CH_2, 1H), 4.42 (s, CH_2, 2H), 4.72 (d, =CH_2, 1H),, 7.15-7.26 (m, Ar-H, 4H); ^{13}C NMR (125 MHz, $CDCl_3$): δ = 47.1, 47.4, 55.6, 86.8,

114.5, 127.2, 129.8, 148.9, 155.7, 159.8 ppm; Elemental analysis for $C_{12}H_{13}NO_3$: C, 65.70; H, 5.97; N, 6.34; O, 21.86. Found C, 65.74; H, 5.98; N, 6.39; O, 21.89.

3-(4-fluorobenzyl)-5-methyleneoxazolidin-2-one (Table 2, entry 5): White liquid, 2.03 g, 98%; ^{1}H NMR (400 MHz, $CDCl_3$) (*δ*/ppm): 4.02 (m, CH_2, 2H), 4.25 (d, =CH_2, 1H), 4.45 (s, CH_2, 2H), 4.74 (d, =CH_2, 1H), 7.04-7.08 (m, Ar-H, 2H), 7.23-7.27 (m, Ar-H, 2H); ^{13}C NMR (125 MHz, $CDCl_3$): δ = 47.0, 47.3, 86.8, 116.1, 130.2, 130.8, 148.9, 155.8 ppm; Elemental analysis for $C_{11}H_{10}FNO_2$: C, 63.71; H, 4.87; F, 9.14; N, 6.72; O, 15.40. Found C, 63.76; H, 4.86; F, 9.17; N, 6.76; O, 15.44.

3-(4-chlorobenzyl)-5-methyleneoxazolidin-2-one (Table 2, entry 6) [12]: White liquid, 2.14 g, 96%; ^{1}H NMR (400 MHz, $CDCl_3$) (*δ*/ppm): 4.03 (m, CH_2, 2H), 4.26 (d, =CH_2, 1H), 4.44 (s, CH_2, 2H), 4.76 (d, =CH_2, 1H), 7.20-7.23 (m, Ar-H, 2H), 7.32-7.35 (m, Ar-H, 2H); ^{13}C NMR (125 MHz, $CDCl_3$): δ = 47.1, 47.2, 87.1, 129.3, 129.6, 133.8, 134.4, 148.9, 155.7 ppm; Elemental analysis for $C_{11}H_{10}ClNO_2$: C, 59.04; H, 4.52; Cl, 15.81; N, 6.24; O, 14.28. Found C, 59.07 ; H, 4.51; Cl, 15.85; N, 6.26; O, 14.31.

5-methylene-3-(4-(trifluoromethyl)benzyl)oxazolidin-2-one (Table 2, entry 7) [12]: White solid, 2.42 g, 94%, m.p. 78.2-80.1 °C;; ^{1}H NMR (400 MHz, $CDCl_3$) (*δ*/ppm): 3.99 (m, CH_2, 2H), 4.22 (d, =CH_2, 1H), 4.46 (s, CH_2, 2H), 4.71 (d, =CH_2, 1H), 7.31-7.35 (m, Ar-H, 2H), 7.53-7.57 (m, Ar-H, 2H); ^{13}C NMR (125 MHz, $CDCl_3$): δ = 47.3, 47.5, 87.5, 124.1, 125.9, 128.6, 130.7, 139.2, 148.8, 155.9 ppm; Elemental analysis for $C_{12}H_{10}F_3NO_2$: C, 56.02; H, 3.91; F, 22.12; N, 5.41; O, 12.38. Found C, 56.04; H, 3.92; F, 22.16; N, 5.45; O, 12.44.

5-methylene-3-(thiophen-2-ylmethyl)oxazolidin-2-one (Table 2, entry 8) [17]: White liquid, 1.89 g, 97%; ^{1}H NMR (400 MHz, $CDCl_3$) (*δ*/ppm): 4.07 (m, CH_2, 2H), 4.25 (d, =CH_2, 1H), 4.61 (s, CH_2, 2H), 4.72 (d, =CH_2, 1H), 6.97-7.02 (m, thiophene-H, 2H), 7.26-7.28 (m, thiophene-H, 1H); ^{13}C NMR (125 MHz, $CDCl_3$): δ = 42.4, 47.3, 87.1, 126.5, 127.2, 127.7, 137.3, 148.8, 155.5 ppm; Elemental analysis for $C_9H_9NO_2S$: C, 55.32; H, 4.67; N, 7.13; O, 16.36; S, 16.37. Found C, 55.37; H, 4.65; N, 7.17; O, 16.39; S, 16.42.

3-butyl-5-methyleneoxazolidin-2-one (Table 2, entry 9) [17]: ^{1}H NMR (400 MHz, $CDCl_3$) (*δ*/ppm): White liquid, 1.43 g, 92%; 0.95 (t, CH_3, 3H), 1.35 (m, CH_2, 2H), 1.56 (m, CH_2, 2H), 3.32 (t, CH_2, 2H), 4.16 (s, CH_2, 2H), 4.28 (d, =CH_2, 1H), 4.74 (d, =CH_2, 1H); ^{13}C NMR (125 MHz, $CDCl_3$): δ = 13.8, 19.7, 29.5, 43.6, 47.8, 86.6, 149.5, 155.8 ppm; Elemental analysis for $C_8H_{13}NO_2$: C, 61.86; H, 8.43; N, 9.01; O, 20.57. Found C, 61.91; H, 8.44; N, 9.03; O, 20.62.

3-cyclohexyl-5-methyleneoxazolidin-2-one (Table 2, entry 10) [18]: White liquid, 1.63 g, 90%; ^{1}H NMR (400 MHz, $CDCl_3$) (*δ*/ppm): 1.10-1.12 (m, 1H), 1.31-1.41 (m, CH_2CH_2, 4H), 1.56-1.58 (m, 1H), 1.71-1.82 (m, CH_2CH_2, 4H), 3.72-3.74 (m, 1H), 4.13 (s, CH_2, 2H), 4.27 (d, =CH_2, 1H), 4.73 (d, =CH_2, 1H); ^{13}C NMR (125 MHz, $CDCl_3$): δ = 25.1, 29.9, 31.3, 36.4, 44.1, 52.3, 77.8, 85.9, 149.8, 154.7, 162.4 ppm; Elemental analysis for $C_{10}H_{15}NO_2$: C, 66.24; H, 8.32; N, 7.68; O, 17.61. Found C, 66.27; H, 8.34; N, 7.73; O, 17.66.

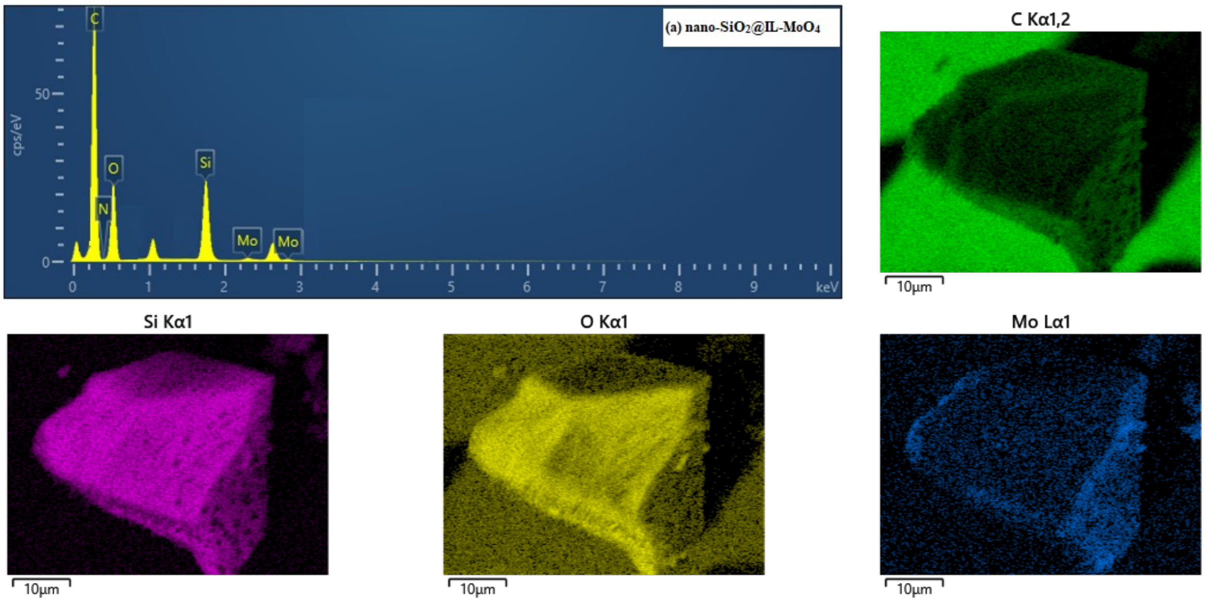

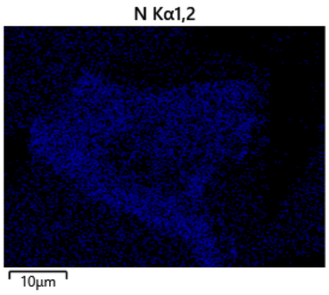
N Kα1,2
10μm

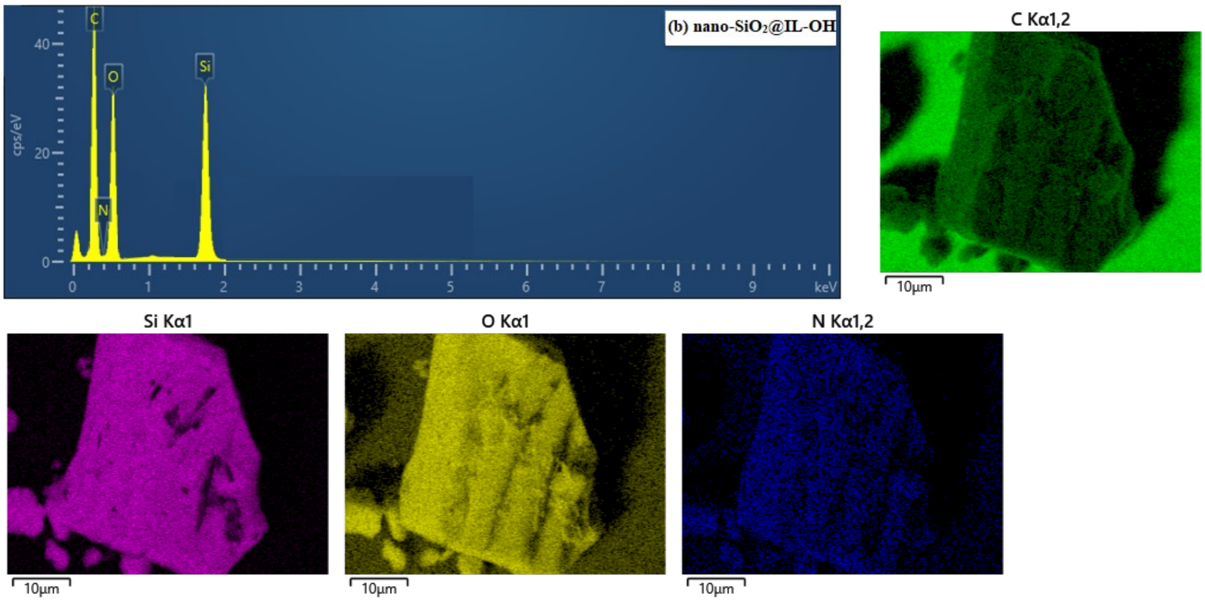
(b) nano-SiO2@IL-OH
cps/eV
keV
C
O
N
Si
C Kα1,2
Si Kα1
O Kα1
N Kα1,2
10μm

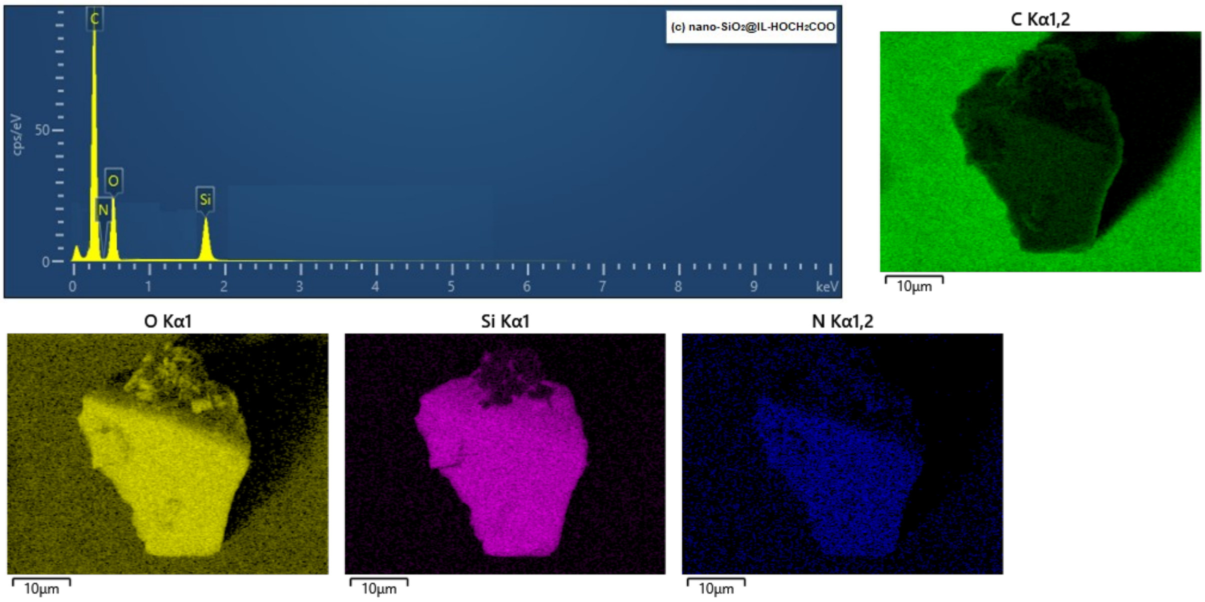
(c) nano-SiO2@IL-HOCH2COO
cps/eV
keV
C
O
N
Si
C Kα1,2
O Kα1
Si Kα1
N Kα1,2
10μm

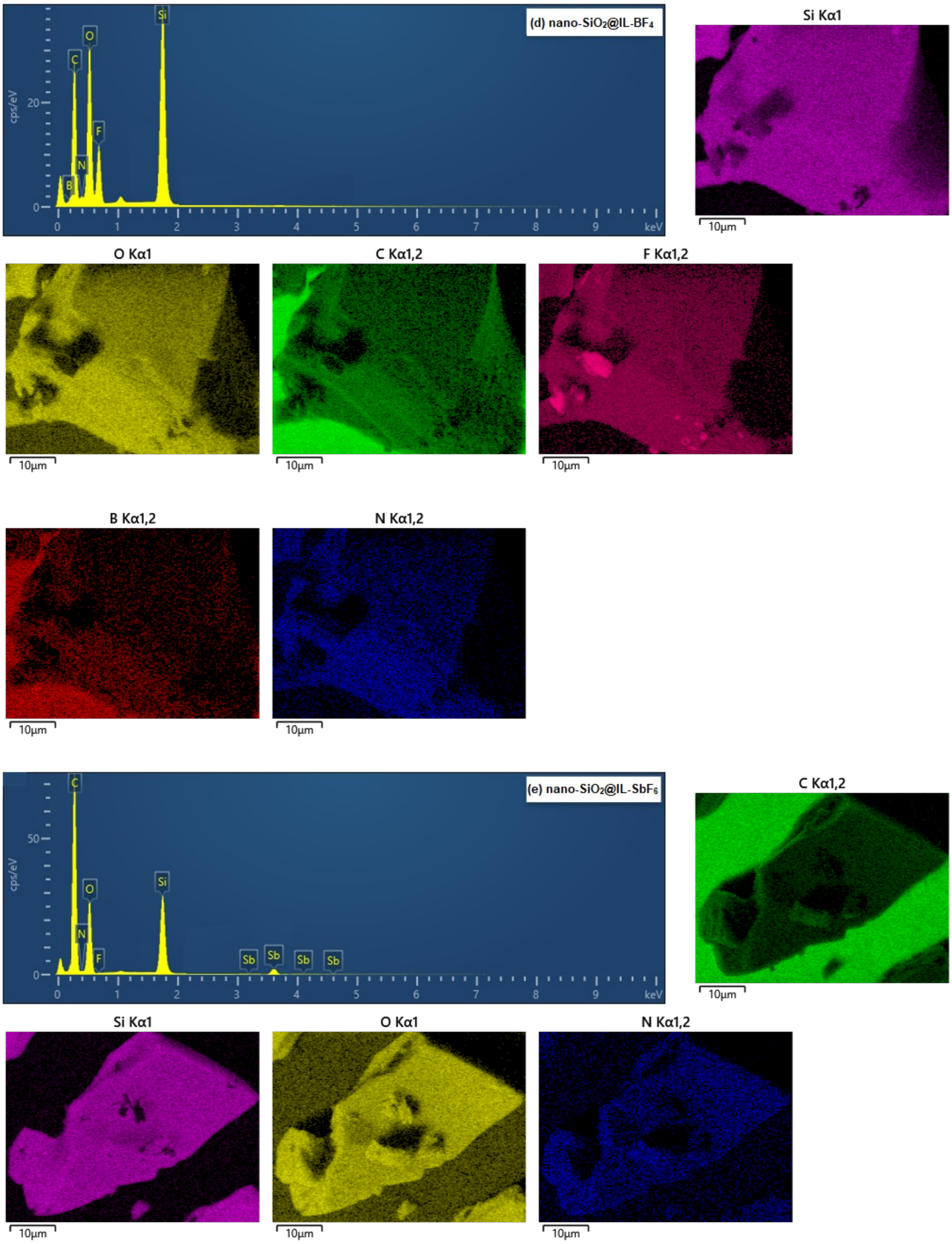
(d) nano-SiO_2@IL-BF_4
cps/eV
keV
Si Kα1
O Kα1
C Kα1,2
F Kα1,2
B Kα1,2
N Kα1,2
10μm
(e) nano-SiO_2@IL-SbF_6
C Kα1,2
Si Kα1
O Kα1
N Kα1,2

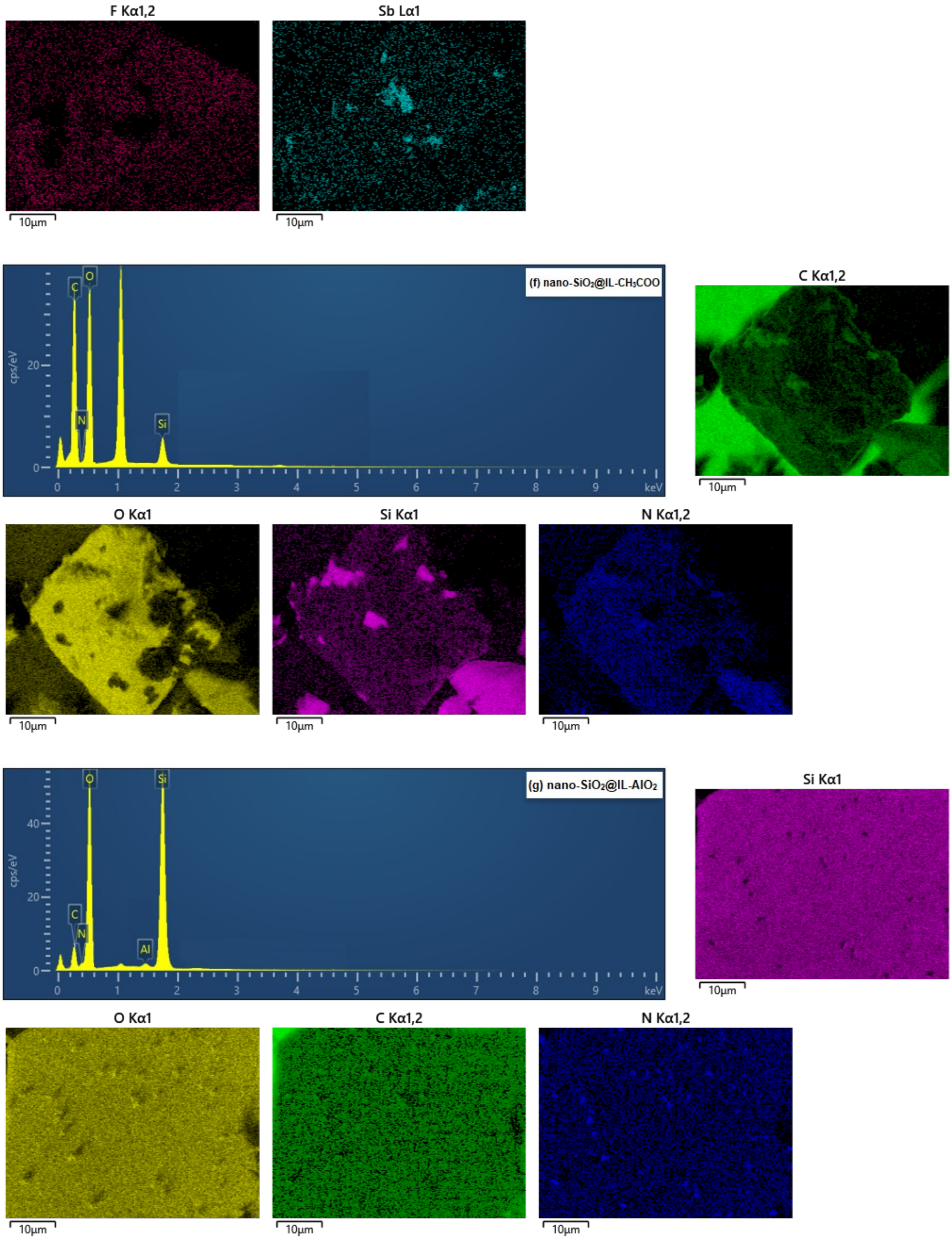
F Kα1,2
Sb Lα1
10μm
(f) nano-SiO₂@IL-CH₃COO
C
O
N
Si
cps/eV
keV
C Kα1,2
O Kα1
Si Kα1
N Kα1,2
(g) nano-SiO₂@IL-AlO₂
Al

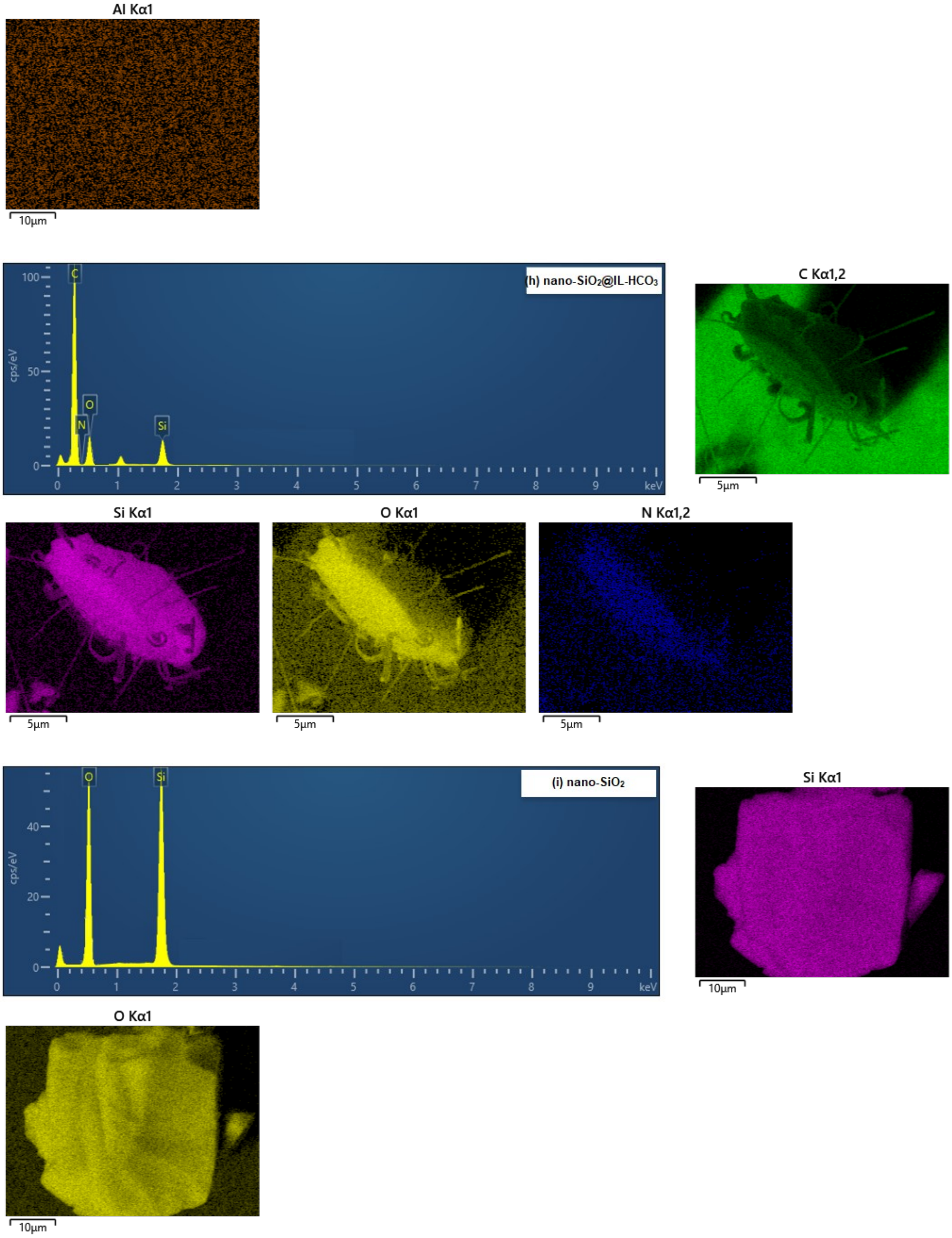

Figure S1. EDS images of (a) IL-MoO_4@nano-SiO_2, (b) IL-OH@nano-SiO_2, (c) IL-$HOCH_2COO$@nano-SiO_2, (d) IL-BF_4@nano-SiO_2, (e) IL-SbF_6@nano-SiO_2, (f) IL-CH_3COO@nano-SiO_2, (g) IL-AlO_2@nano-SiO_2, (h) IL-HCO_3@nano-SiO_2, and (i) nano-SiO_2.

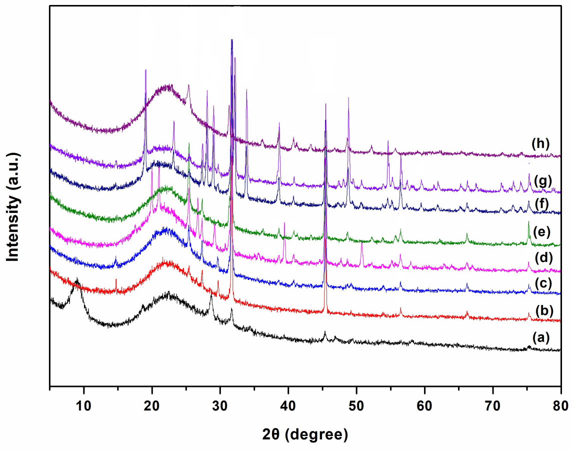

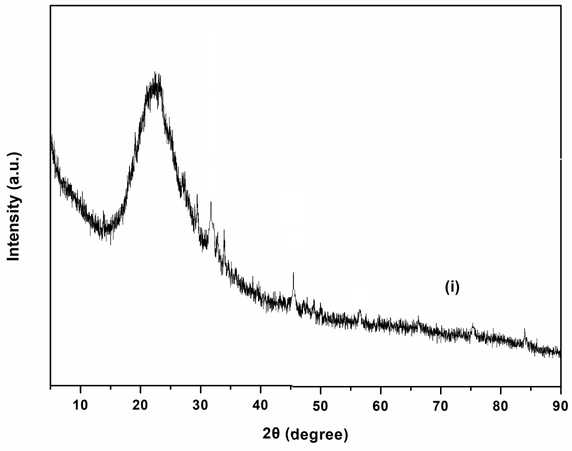

Figure S2. XRD pattern of (a) IL-MoO_4@nano-SiO_2, (b) IL-OH@nano-SiO_2, (c) IL-$HOCH_2COO$@nano-SiO_2, (d) IL-BF_4@nano-SiO_2, (e) IL-SbF_6@nano-SiO_2, (f) IL-CH_3COO@nano-SiO_2, (g) IL-AlO_2@nano-SiO_2, (h) IL-HCO_3@nano-SiO_2, and (i) nano-SiO_2.

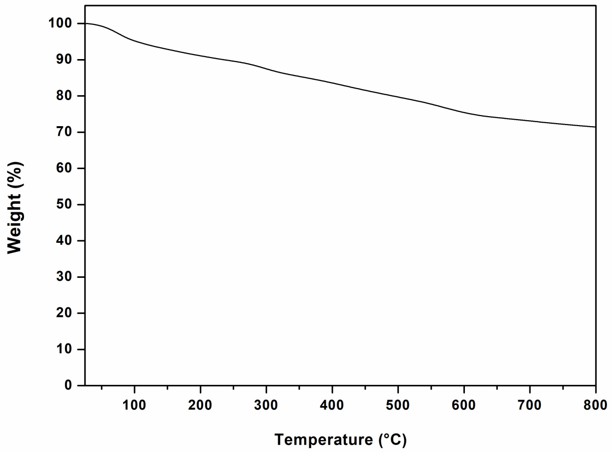

Figure S3. TGA curve of IL-SbF_6@nano-SiO_2 catalyst.

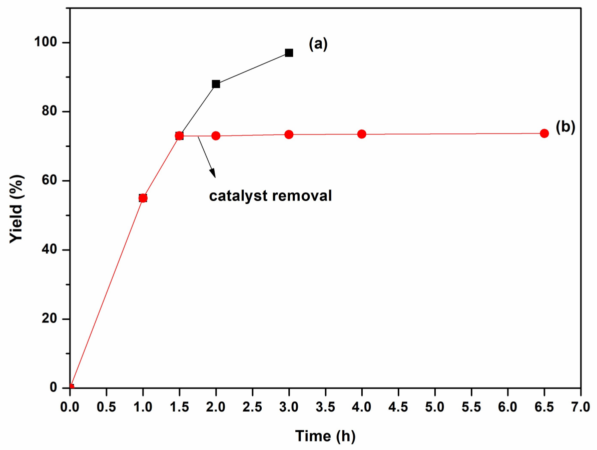

Figure S4. Hot filtration test during the catalytic process. Reaction conditions: *N*-benzylprop-2-yn-1-amine (10 mmol), H_2O (10 mL), CO_2 (0.3 MPa) at 50 °C with catalyst IL-SbF_6@nano-SiO_2 (0.4 g) (a) and catalyst removal (b) after 3 h.

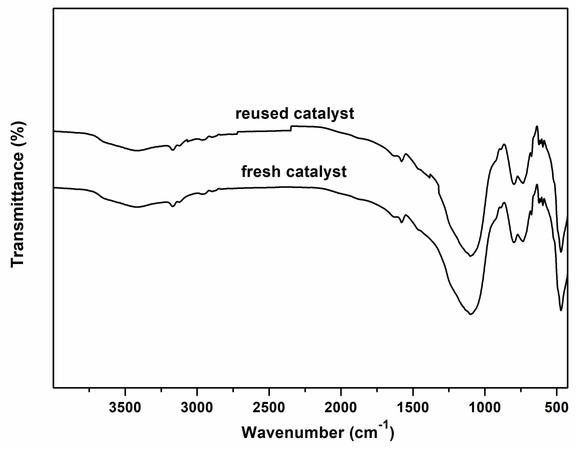

Figure S5. FT-IR spectra of IL-SbF_6@nano-SiO_2 catalyst before and after reaction.

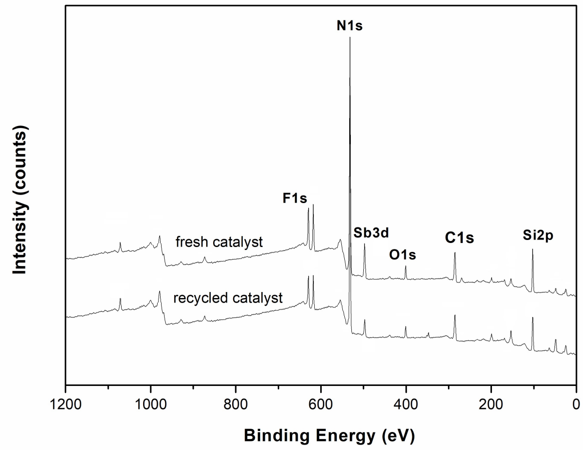

Figure S6. XPS spectra of fresh and reused IL-SbF_6@nano-SiO_2 catalyst.

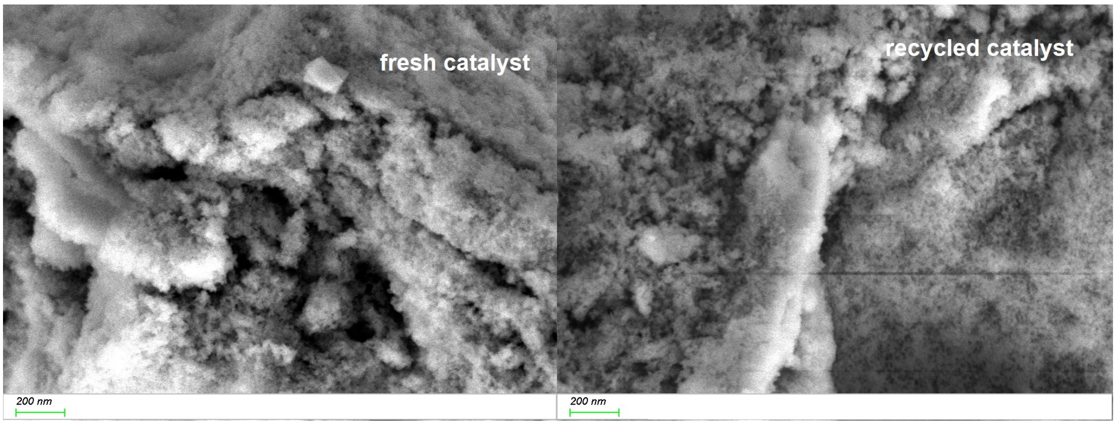

Figure S7. SEM images of fresh and six times recycled IL-SbF_6@nano-SiO_2 catalyst.

Copies of ^{1}H NMR and ^{13}C NMR spectra

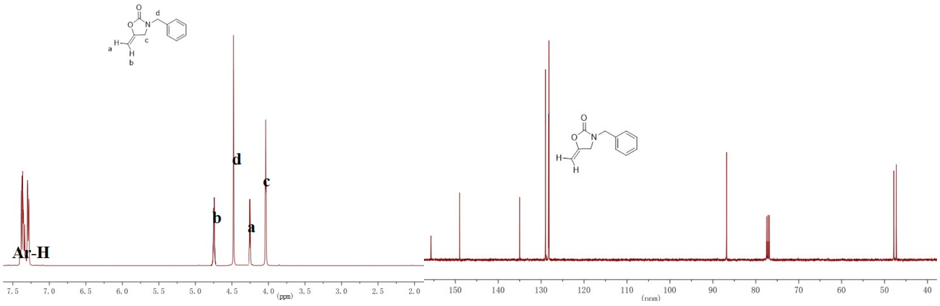

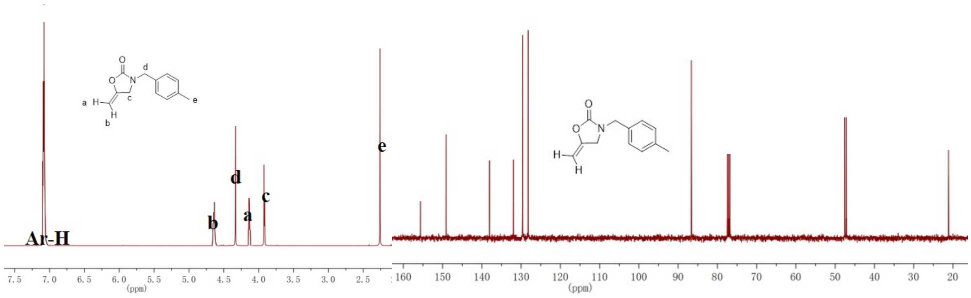

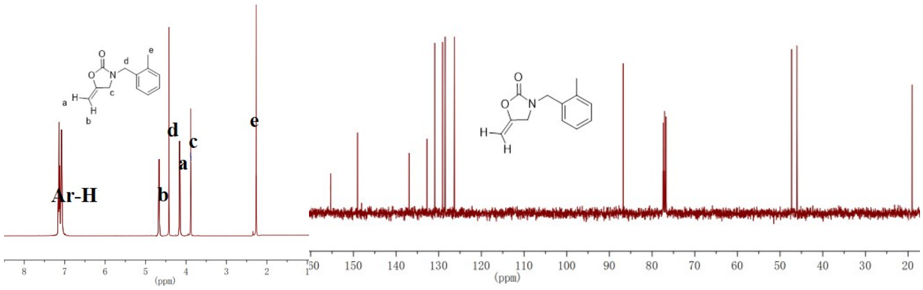

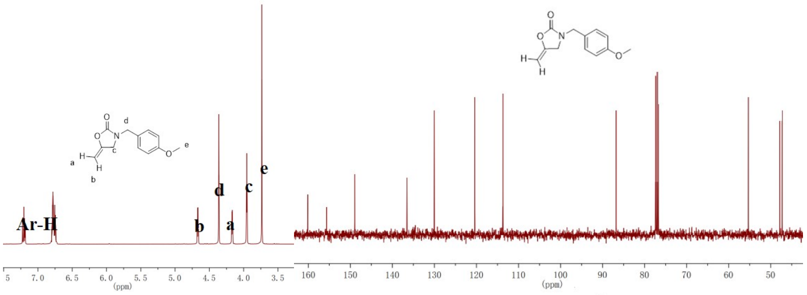

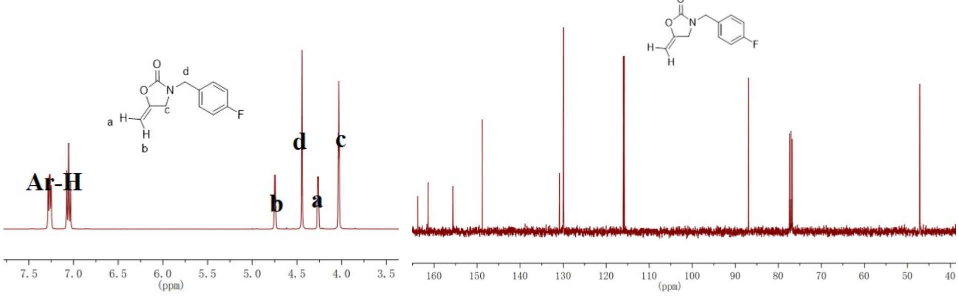

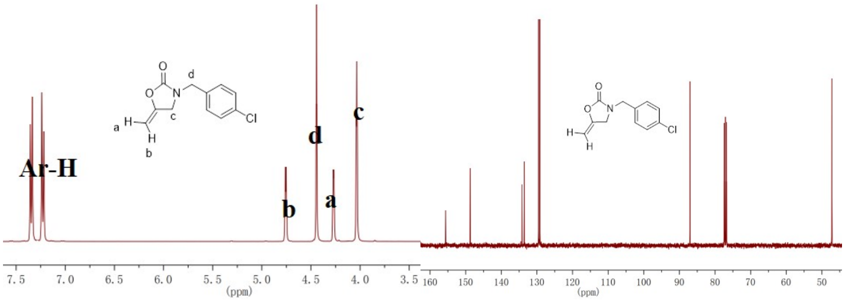
Ar-H
a
b
c
d
Cl
(ppm)

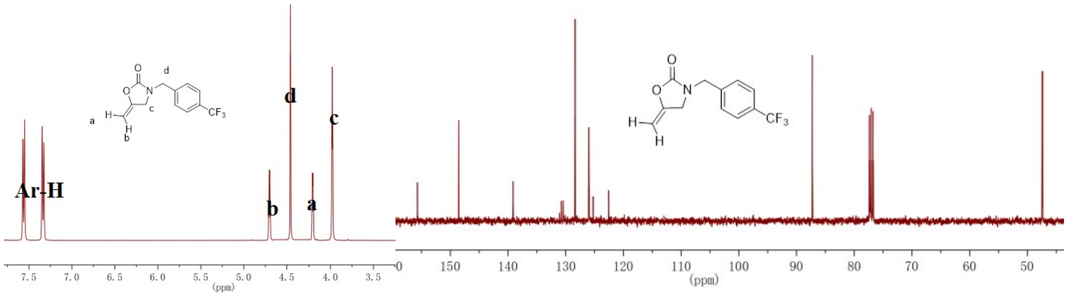
Ar-H
a
b
c
d
CF_3
(ppm)

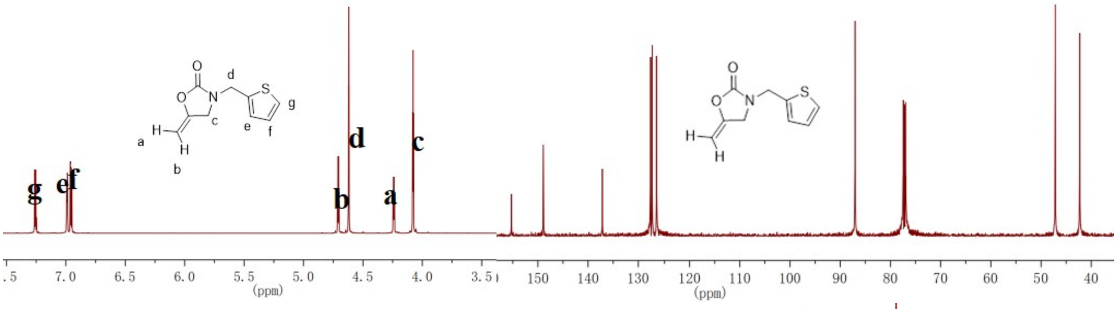
g
e
f
b
d
a
c
S
(ppm)

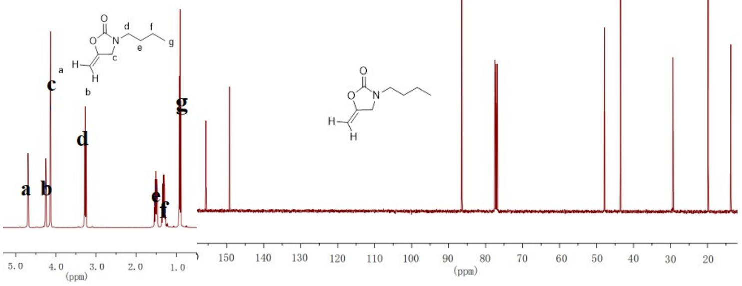
a
b
c
d
e
f
g
(ppm)

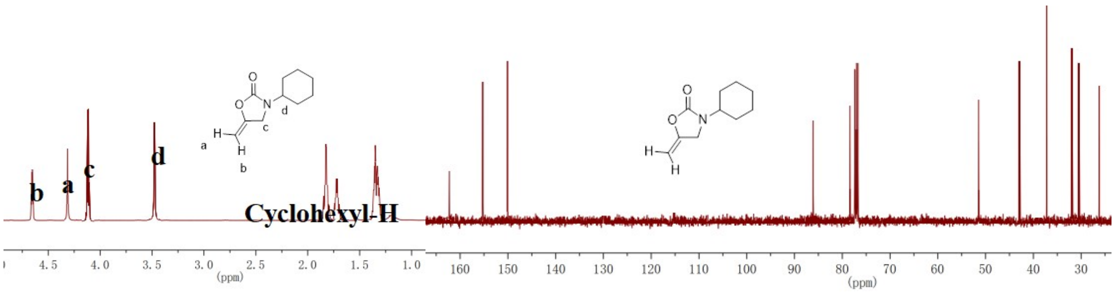
b
a
c
d
Cyclohexyl-H
(ppm)
